# 

**Published:** 1913-11-29

**Authors:** 


### LEICESTER ROYAL INFIRMARY.

On the attainment of his eightieth birthday, Mr. Thomas Sharland, of Leicester, sent a cheque for £100 to Sir Edward Wood for the funds of the Royal Infirmary. In sending the contribution, Mr. Sharland intimates that he has been saving the amount for the last nine years, his desire to celebrate his eightieth birthday being stimulated by a similar gift from the late Dr. Nuttall in 1905.

### Appointments.

Mr. H. Morriston Davies, F.R.C.S., has been appointed honorary surgeon to the City of London Hospital for Diseases of the Chest, Victoria Park, E.

North Darley Urban Council has appointed Dr. Morton as medical officer of health, in place of Dr. H. Moxon, resigned. Dr. Morton was house surgeon at the Sheffield Royal Infirmary from 1893 to 1896, and from the latter year until 1911. He was also honorary surgeon to the Sheffield Children's Hospital.

The following appointments have been made at the Manchester Royal Infirmary : Honorary consulting dental surgeon, Mr. W. A. Hooton, M.R.C.S. (Eng.), L.R.C.P. (Lond.), L.D.S.R.C.S. (Eng.); acting dental surgeon, Mr. E. C. Long, L.R.D.R.C.S. (Eng.); surgical registrar, Mr. John Gow, M.B., Ch.B. (Vict.); house surgeon to special departments, Mr. G. B. Jameson, M.R.C.S. (Eng.), L.R.C.P. (Lond.); house physicians, Mr. A. V. Stocks, B.A. (Cantab.), M.B., Ch.B. (Vict.), Mr. F. A. Bearn, M.B., Ch.B. (Vict.), Mr. F. C. Bentz, M.B., Ch.B. (Vict.); senior house surgeons, Mr. H. D. Willis, M.B., Ch.B. (Vict.), L.M.S.S.A., Mr. J. G. Bennett, M.B., Ch.B. (Vict.); junior house surgeons, Mr. C. H. Crawshaw, M.R.C.S. (Eng.), L.R.C.P. (Lond.), Mr. H. F. Hutchinson, M.B., Ch.B. (Vict.), M.R.C.S., L.R.C.P.

THE FORTUNE OF A MEDICAL INVENTOR.

Dr. Frederick William Forbes Ross, M.D., F.R.C.S., D.P.H., of New Cavendish Street, W., inventor of several surgical instruments, who died intestate, left estate of the gross value of £1,077, of which the net personalty has been sworn at £523.

### Gifts and Bequests.

Mr. Edward Storey, of Lancaster, has left £500 to the Royal Lancaster Infirmary.

Mr. Rodie Macfee, of Liverpool, fruit broker, has bequeathed £1,500 to endow a "Rodie Macfee Bed" in the Liverpool Infirmary for Children; £1,500 to endow a "Jane Brownlie Macfee Bed" in the Hospital for Women, Liverpool; and £600 to endow a "Rodie Macfee Bed" in the Children's Convalescent Home, West Kirby.

Mr. Sophus Simmelkjoer, of Gower Street, W.C., civil engineer, a Danish subject who had become a domiciled Englishman, has bequeathed, subject to bequests to members of his family and intimate friends, amounting to about £25,000, the residue of his property for division between the Middlesex Hospital, the Westminster Hospital, "the Northern Hospital, Holloway, N.," St. Saviour's Hospital, Osnaburgh Street, N.W., and two other charities. It is stated that the gross value of this residuary bequest will be about £15,000.

Mr. George Anderson, of Upper Norwood, gas engineer, who left net personalty £85,085, has bequeathed £500 (payable by five equal annual instalments) to the National Anti-Vivisection Society; £200 each to the Lock Hospital for Women, the Caledonian Asylum, Holloway, and the Scottish Hospital, Crane Court, E.C., subject to the condition as regards the hospitals that they shall certify that they do not practise or sanction vivisection, either directly or through medical schools attached to them, and failing such certificate the legacy to such hospital is to be reduced to £100.

### £63,000 FOR CHARITIES.

Mr. William Weir, of Kildonan, chief partner of Messrs. Baird, ironmasters, Glasgow, whose estate is expected to exceed £1,000,000, has bequeathed £63,000 for charitable purposes, free of duty. He left £10,000 each to the Glasgow Royal Infirmary and the Glasgow Western Infirmary; £5,000 each to Glasgow Victoria Infirmary and the University of Glasgow for additional assistant to the Professor of Materia Medica; £2,000 each to the Kilmarnock Infirmary and the Ayr Infirmary; £500 each to the Glasgow Eye Infirmary and the Glasgow Ophthalmic Institution; and £10,000 to other charitable and benevolent institutions in Glasgow in the discretion of his trustees.

